# Association between fluid balance and mortality for heart failure and sepsis: a propensity score-matching analysis

**DOI:** 10.1186/s12871-022-01865-5

**Published:** 2022-10-22

**Authors:** Bufan Zhang, Shaohua Guo, Zean Fu, Naishi Wu, Zhigang Liu

**Affiliations:** 1 Department of Cardiovascular Surgery, Tianjin Medical University General Hospital, Tianjin, People’s Republic of China; 2 Department of Cardiovascular Surgery & Intensive Care Unit, TEDA International Cardiovascular Hospital, Cardiovascular Clinical College of Tianjin Medical University, Tianjin, People’s Republic of China; 3 Tianjin Key Laboratory of Ionic-Molecular Function of Cardiovascular Disease, Department of Cardiology, Tianjin Institute of Cardiology, Second Hospital of Tianjin Medical University, Tianjin, People’s Republic of China

**Keywords:** Critically ill patients, Heart failure, Sepsis, Fluid balance, In-hospital mortality

## Abstract

**Background:**

Fluid resuscitation is necessary to correct the sepsis-induced hypoperfusion, which is contradictory to the treatment of heart failure. This study explored the association between fluid balance (FB) of the first 24 h after ICU admission and mortality in critically ill patients with heart failure and sepsis.

**Methods:**

Data were extracted from the Medical Information Mart for Intensive Care database. The locally weighted scatterplot smoothing (Lowess) method was used to demonstrate the relationship between FB and in-hospital mortality. Groups were divided into high FB (≥ 55.85 ml/kg) and low FB (< 55.85 ml/kg) according to the cut-off value of FB using Receiver operating characteristic analysis and Youden index method. The primary outcome was in-hospital mortality. Subgroup analyses, multivariable logistic regression analyses, and Kaplan-Meier curves were used to detect the association and survival difference between groups. Inverse probability treatment weighting (IPTW) and propensity score matching (PSM) were performed to minimize the bias of confounding factors and facilitate the comparability between groups.

**Results:**

A total of 936 patients were included. The Lowess curve showed an approximate positive linear relationship for FB and in-hospital mortality. In the multivariable logistic regression adjusted model, high FB showed strong associations with in-hospital mortality (OR 2.53, 95% CI 1.60–3.99, p < 0.001) as compared to the low FB group. In IPTW and PSM models, high FB consistently showed higher in-hospital mortality (IPTW model: OR 1.94, 95% CI 1.52–2.49, p < 0.001; PSM model: OR 2.93, 95% CI 1.75–4.90, p < 0.001) and 30-day mortality (IPTW model: OR 1.65, 95% CI 1.29–2.10, p < 0.001; PSM model: OR 2.50, 95% CI 1.51–4.15, p < 0.001), compared with the low FB group.

**Conclusion:**

For critically ill patients with heart failure and sepsis, high FB within the first 24 h after ICU admission could serve as an independent risk factor for in-hospital mortality and 30-day mortality. The avoidance of fluid overload exerts important effects on reducing mortality in such patients.

**Supplementary Information:**

The online version contains supplementary material available at 10.1186/s12871-022-01865-5.

## Background

Heart failure, as an increasing global prevalent disease, is featured as cardiovascular dysfunction with high morbidity and mortality [1]. More than 64 million people worldwide suffer from heart failure, which seriously affects their quality of life, especially for the elderly [2]. Among these patients, chronic heart failure is more common than new-onset heart failure. Infection is a common precipitating factor to induce acute decompensation in chronic heart failure. The OFICA study published by Logeart et al. demonstrated over a quarter of acute heart failure patients were induced by infection [3]. Furthermore, an Israeli study showed 38% of patients with heart failure were hospitalized because of infection-related factors [4]. Sepsis is regarded as a serious and life-threatening disease featured with multiple organ dysfunction due to patients’ dysregulation to infection. Thus, heart failure with sepsis has a complicated pathophysiological mechanism, with high mortality and a high probability of being admitted into the intensive care unit (ICU). The treatment for these patients is full of challenge since the therapeutic strategies for sepsis and heart failure are contradictory. Fluid resuscitation, as an important treatment for early management, is necessary to resolve the tissue and organ hypoperfusion induced by sepsis according to the Surviving Sepsis Campaign guidelines [5, 6]. However, excessive fluid intake can worsen the congestive symptom and increase the risk of poor prognosis for heart failure patients. Current strategies are empirical, and no relevant guidelines individually provide specific approaches according to various haemodynamic states [7]. There still remained unclear on patients’ management for fluid balance status. Therefore, this study mainly discussed the association between fluid balance (FB) of the first 24 h after ICU admission and mortality in critically ill patients with heart failure and sepsis.

## Materials and methods

### Data source

All the data were extracted from Medical Information Mart for Intensive Care III (MIMIC III version 1.4) database, a free-available database containing more than 40,000 patients in the intensive care unit (ICU) of the Beth Israel Deaconess Medical Center [8]. All the information in this database was anonymized to protect patients’ privacy so the ethical approval statement and informed consent were not required. One author (certification number 38,653,219 for author Zhang) gained access to the database and extracted the data using PostgreSQL tools version 10.

### Study population and outcome

Sepsis patients were retrieved in terms of sepsis-3 criteria: (1) patients were confirmed with infection by positive results of microbial culture; (2) Sequential Organ Failure Assessment (SOFA) score ≥ 2 [9]. Heart failure patients were identified using the International Classification of Diseases (ICD) 9 code, including 398.91, 402.01, 402.11, 402.91, 404.01, 404.03, 404.11, 404.13, 404.91, 404.93, 428.XX. We classified heart failure as heart failure with reduced ejection fraction (LVEF ≤ 40%), heart failure with mildly-reduced ejection fraction (LVEF 40 − 50%), and heart failure with preserved ejection fraction (LVEF ≥ 50%) according to left ventricular ejection fraction (LVEF) [10]. For this study, only the first ICU admission was included in this study if patients were admitted in ICU more than once. Patients younger than 18 years or without sufficient data to calculate fluid balance were excluded. In addition, patients who spent less than 24 h in ICU, or underwent renal replacement therapy or cardiac surgery were excluded.

The outcomes were also extracted, including in-hospital mortality, 30-day mortality, as well as length of stay (LOS) in ICU and hospital. In-hospital mortality was defined as the primary outcome. The secondary outcomes were 30-day mortality, as well as LOS in ICU and hospital.

### Data extraction

The following data were collected at the first ICU admission as baseline characteristics: age, gender, weight, ethnicity, infection site, left ventricular ejection fraction, mean arterial pressure (MAP). Laboratory parameters included white blood cell, hemoglobin, pH, serum potassium, serum sodium, serum bicarbonate, serum chloride, serum lactate, serum creatinine, Troponin T, and N-terminal pro-B-type natriuretic peptide (NT-proBNP). Medications included angiotensin-converting enzyme inhibitor/angiotensin receptor blocker (ACEI/ARB), beta-blocker, and vasopressor. The severity of illness was evaluated by sequential organ failure assessment (SOFA) score. Comorbidities included coronary heart disease, hypertension, chronic obstructive pulmonary disease (COPD), chronic kidney disease (CKD), cirrhosis, and diabetes. Vasopressor included epinephrine, norepinephrine, vasopressin, dopamine, dobutamine, and phenylephrine. Fluid intake and output were recorded at the first 24 h after ICU admission. Fluid intake included crystalloid, colloid, and blood products. Fluid output was defined as urine output, drainage fluid from chest and cerebral tubes, and stool. We calculated the difference between fluid input and fluid output as fluid balance (FB). All the fluid volumes have been corrected for each individual’s body weight. All baseline data were included within 24 h after ICU admission.

### Statistical analysis

Categorical data were recorded as number and percentage, and compared using Pearson’s chi-squared test or Fisher’s exact test. Continuous data were recorded as mean ± standard deviation (mean ± SD) or median with interquartile range (IQR), and compared using Student’s t test or Wilcoxon rank-sum test as appropriate. The missing values of all the included variables were less than 30% and filled by the multiple imputation method (details illustrated in Supplement Table S1). The locally weighted scatterplot smoothing (Lowess) method was used to demonstrate the crude relationship between FB and in-hospital mortality. Receiver operating characteristic analysis and Youden index method were used to calculate the cut-off value of FB. For a better explanation, groups were divided according to the cut-off value and analyzed by univariable and multivariable logistical regression. Subgroup analysis was performed to detect the association of FB and in-hospital mortality among various groups according to median age, gender, LVEF, median MAP, median SOFA score, median serum lactate level, median NT-proBNP level, coronary heart disease, hypertension, COPD, CKD, cirrhosis, and diabetes. Inverse probability treatment weighting (IPTW) [11] and propensity score matching (PSM) [12] were performed to minimize the bias of confounding factors and facilitate the comparability between groups. The ratio was set as 1:1 match with a caliper width of 0.05 in the PSM model. The Kaplan-Meier method with log-rank test was performed to compare the 30-day survival rates between high and low FB. The data cleaning, statistical analyses, and data visualizations were performed using Stata version 16.0, and R software version 4.0.2. P value < 0.05 with a two-sided test was regarded as statistical significance.

## Results

### Baseline characteristics

**Fig. 1:**
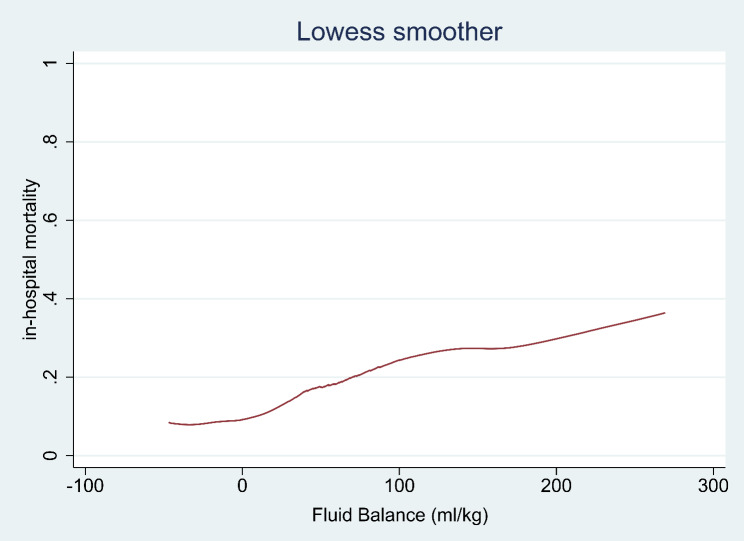
Association of fluid balance and in-hospital mortality

A total of 936 patients were included in our study, and the selection process was described in Supplement Figure S1. The mean age of included patients was 71.59 ± 14.27 years and 52.24% were male. The median (IQR) LOS in ICU and hospital were respectively 3.34 (1.93 to 7.52) and 9.63 (5.77 to 17.05) days. The in-hospital and 30-day mortality reached 16.77% and 17.74%. The Lowess curve displayed an approximate positive linear relationship for FB and in-hospital mortality (Fig. 1). For further analysis, the cut-off value (55.85 ml/kg) of FB was calculated, and groups were further divided into the high FB group (≥ 55.85 ml/kg) and the low FB group (< 55.85 ml/kg), which was presented in Table 1.

**Table 1:** Baseline characteristics between high and low FB groups

Variables	Total (n = 936)	High FB (n = 349)	Low FB (n = 587)	p
Age (years)	71.59 ± 14.27	73.37 ± 14.65	70.54 ± 13.94	0.003
Gender, male, n (%)	489 (52.24)	173 (49.57)	316 (53.83)	0.207
Weight (kg)	81.03 ± 24.03	75.02 ± 19.93	84.60 ± 25.52	< 0.001
Ethnicity, n (%)				0.112
White	696 (74.36)	270 (77.36)	426 (72.57)	
Black	103 (11.00)	29 (8.31)	74 (12.61)	
Other	137 (14.64)	50 (14.33)	87 (14.82)	
Infection site, n (%)				0.617
Blood	652 (69.66)	243 (69.63)	409 (69.68)	
Urine	187 (19.98)	66 (18.91)	121 (20.61)	
Other	97 (10.36)	40 (11.46)	57 (9.71)	
LVEF, n (%)				0.556
≤ 40%	260 (27.78)	90 (25.79)	170 (28.96)	
40–50%	78 (8.33)	31 (8.88)	47 (8.01)	
≥ 50%	598 (63.89)	228 (65.33)	370 (63.03)	
MAP (mm Hg)	77.30 ± 17.94	75.47 ± 17.38	78.39 ± 18.19	0.016
Laboratory test				
White blood cell (10^9^/L)	10.3 (7.1, 15.55)	11.2 (7, 16.7)	10.1 (7.2, 14.8)	0.046
Hemoglobin (g/dL)	11.46 ± 2.01	11.48 ± 2.04	11.45 ± 1.99	0.831
pH	7.36 ± 0.10	7.35 ± 0.11	7.37 ± 0.10	0.005
Serum potassium (mmol/L)	4.38 ± 0.87	4.38 ± 0.92	4.39 ± 0.85	0.867
Serum sodium (mmol/L)	137.77 ± 5.05	137.79 ± 5.58	137.76 ± 4.70	0.913
Serum bicarbonate (mmol/L)	24.35 ± 5.20	23.07 ± 5.10	25.11 ± 5.12	< 0.001
Serum chloride (mmol/L)	101.65 ± 6.29	102.56 ± 6.73	101.10 ± 5.95	< 0.001
Serum lactate (mmol/L)	1.9 (1.3, 2.8)	2.1 (1.4, 3.2)	1.8 (1.3, 2.5)	< 0.001
Serum creatinine (mg/dL)	1.3 (0.9, 2.1)	1.3 (0.9, 2.1)	1.3 (0.9, 2)	0.608
Troponin T (ng/mL)	0.04 (0.02, 0.11)	0.04 (0.02, 0.08)	0.04 (0.02, 0.12)	0.983
NT-proBNP (pg/mL)	5076 (1939, 12919.5)	5627 (2044, 11642)	4671 (1870, 13556)	0.439
Drug, n (%)				
ACEI/ARB	112 (11.97)	13 (3.72)	99 (16.87)	< 0.001
Beta-blocker	387 (41.35)	117 (33.52)	270 (46.00)	< 0.001
Vasopressor	368 (39.32)	217 (62.18)	151 (25.72)	< 0.001
SOFA	5 (3, 8)	7 (4, 10)	5 (3, 7)	< 0.001
Comorbidities, n (%)				
Coronary heart disease	437 (46.69)	140 (40.11)	297 (50.60)	0.002
Hypertension	484 (51.71)	181 (51.86)	303 (51.62)	0.942
COPD	71 (7.59)	13 (3.72)	58 (9.88)	0.001
CKD	336 (35.90)	112 (32.09)	224 (38.16)	0.061
Cirrhosis	70 (7.48)	31 (8.88)	39 (6.64)	0.208
Diabetes	443 (47.33)	133 (38.11)	310 (52.81)	< 0.001
Fluid balance status				
Fluid intake (ml/kg/24 h)	61.14 (30.00, 109.78)	127.60 (97.41, 174.30)	36.36 (20.54, 56.19)	< 0.001
Fluid output (ml/kg/24 h)	21.81 (12.07, 37.70)	19.79 (11.11, 37.17)	23.31 (12.66, 38.17)	0.076
Fluid balance (ml/kg/24 h)	36.90 (5.09, 81.19)	98.28 (73.08, 142.88)	13.93 (-5.11, 32.33)	< 0.001
Clinical Outcomes				
In-hospital mortality, n (%)	157 (16.77)	97 (27.79)	60 (10.22)	< 0.001
30-day mortality, n (%)	166 (17.74)	99 (28.37)	67 (11.41)	< 0.001
ICU LOS (days)	3.34 (1.93, 7.52)	4.85 (2.62, 9.08)	2.82 (1.82, 5.91)	< 0.001
Hospital LOS (days)	9.63 (5.77, 17.05)	11.35 (7.32, 21.11)	8.52 (4.97, 14.94)	< 0.001

Abbreviations: ACEI, angiotensin-converting enzyme inhibitor; ARB, angiotensin receptor blocker; CKD, chronic kidney disease; COPD, chronic obstructive pulmonary disease; FB, fluid balance; ICU, intensive care unit; LOS, length of stay; LVEF, left ventricular ejection fraction; MAP, mean arterial pressure; NT-proBNP, N-terminal pro-B-type natriuretic peptide; SOFA, sequential organ failure assessment

### Association between fluid balance and in-hospital mortality

**Fig. 2:**
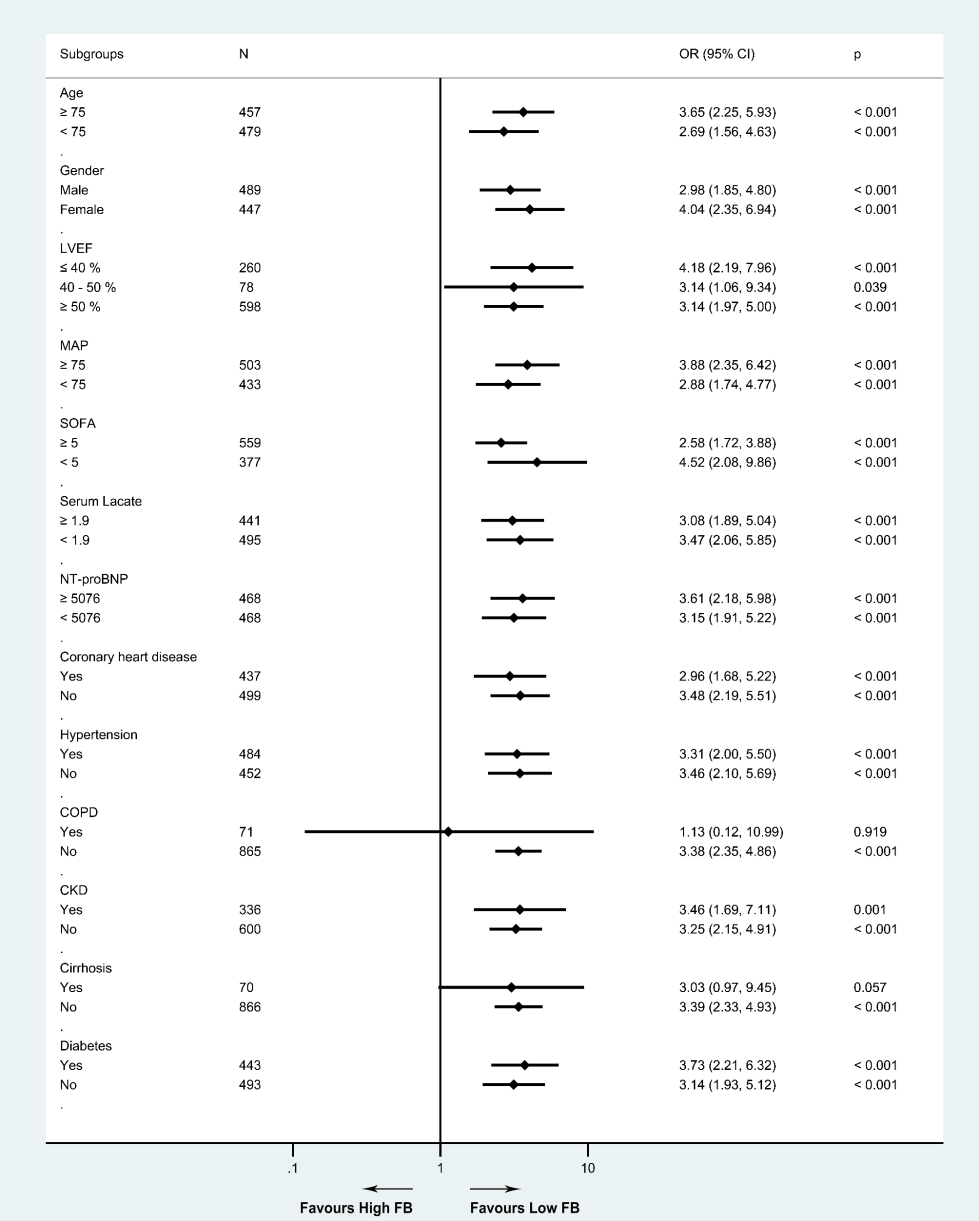
Forrest plots for the association of high FB and clinical outcomes in subgroups. Abbreviations: CI, confidence interval; CKD, chronic kidney disease; COPD, chronic obstructive pulmonary disease; FB, fluid balance; LVEF, left ventricular ejection fraction; MAP, mean arterial pressure; NT-proBNP, N-terminal pro-B-type natriuretic peptide; OR, odds ratio; SOFA, sequential organ failure assessment

After groups were divided according to the cut-off value, the difference of FB between groups was mainly associated with fluid intake (p < 0.001) instead of fluid output (p = 0.076) (Table 1). Patients with high FB showed higher in-hospital mortality (27.79% vs. 10.22%, p < 0.001) and 30-day mortality (28.37% vs. 11.41%, p < 0.001), as well as longer LOS in ICU (4.85 [2.62, 9.08] vs. 2.82 [1.82, 5.91], p < 0.001) and hospital (11.35 [7.32, 21.11] vs. 8.52 [4.97, 14.94], p < 0.001), as compared to the lower FB group (Table 1).The crude model demonstrated that in-hospital mortality (OR 3.38, 95% CI 2.37–4.82, p < 0.001) and 30-day mortality (OR 3.07, 95% CI 2.18–4.34, p < 0.001) were significantly higher for patients with high FB (Table 2). Subgroup analyses revealed that high FB had higher risks of in-hospital mortality in most of the subgroups except for patients with COPD or cirrhosis (Fig. 2). In the adjusted model, which further adjusted for all included covariates, high FB was regarded as an independent risk factor for in-hospital mortality (OR 2.53, 95% CI 1.60–3.99, p < 0.001) and 30-day mortality (OR 2.08, 95% CI 1.34–3.23, p = 0.001) compared with low FB (Table 2). FB also showed strong and consistent associations with in-hospital mortality (crude model: OR 1.67, 95% CI 1.39–1.99 per SD increase, p < 0.001; adjusted model: OR 1.32, 95% CI 1.04 to 1.69 per SD increase, p = 0.024) and 30-day mortality (crude model: OR 1.67, 95% CI 1.40–1.99 per SD increase, p < 0.001; adjusted model: OR 1.28, 95% CI 1.01–1.63 per SD increase, p = 0.041) when analyzed as a continuous variable (Table 2).

**Table 2:** Association of FB and mortality in various models

	Crude Model		Adjusted Model		IPTW Model		PSM Model	
	OR (95% CI)	p	OR (95% CI)	p	OR (95% CI)	p	OR (95% CI)	p
Categorical FB								
In-hospital mortality								
Low FB	1.0 (Reference)	-	1.0 (Reference)	-	1.0 (Reference)	-	1.0 (Reference)	-
High FB	3.38 (2.37, 4.82)	< 0.001	2.53 (1.60, 3.99)	< 0.001	1.94 (1.52, 2.49)	< 0.001	2.93 (1.75, 4.90)	< 0.001
30-day mortality								
Low FB	1.0 (Reference)	-	1.0 (Reference)	-	1.0 (Reference)	-	1.0 (Reference)	-
High FB	3.07 (2.18, 4.34)	< 0.001	2.08 (1.34, 3.23)	0.001	1.65 (1.29, 2.10)	< 0.001	2.50 (1.51, 4.15)	< 0.001
Continuous FB								
In-hospital mortality	1.67 (1.39, 1.99)	< 0.001	1.32 (1.04, 1.69)	0.024	1.66 (1.39, 1.99)	< 0.001	1.45 (1.13, 1.88)	0.004
30-day mortality	1.67 (1.40, 1.99)	< 0.001	1.28 (1.01, 1.63)	0.041	1.67 (1.40, 1.99)	< 0.001	1.35 (1.04, 1.74)	0.022

Note: Crude model: No covariates were adjusted. Adjusted Model: adjusted for age, gender, ethnicity, weight, infection site, LVEF, MAP, laboratory test, drug, SOFA, and comorbidities

Abbreviations: CI, confidence interval; FB, fluid balance; LVEF, left ventricular ejection fraction; IPTW, inverse probability of treatment weighting; MAP, mean arterial pressure; OR, odds ratio; PSM, propensity score-matching; SOFA, sequential organ failure assessment

### IPTW and PSM analyses

**Fig. 3:**
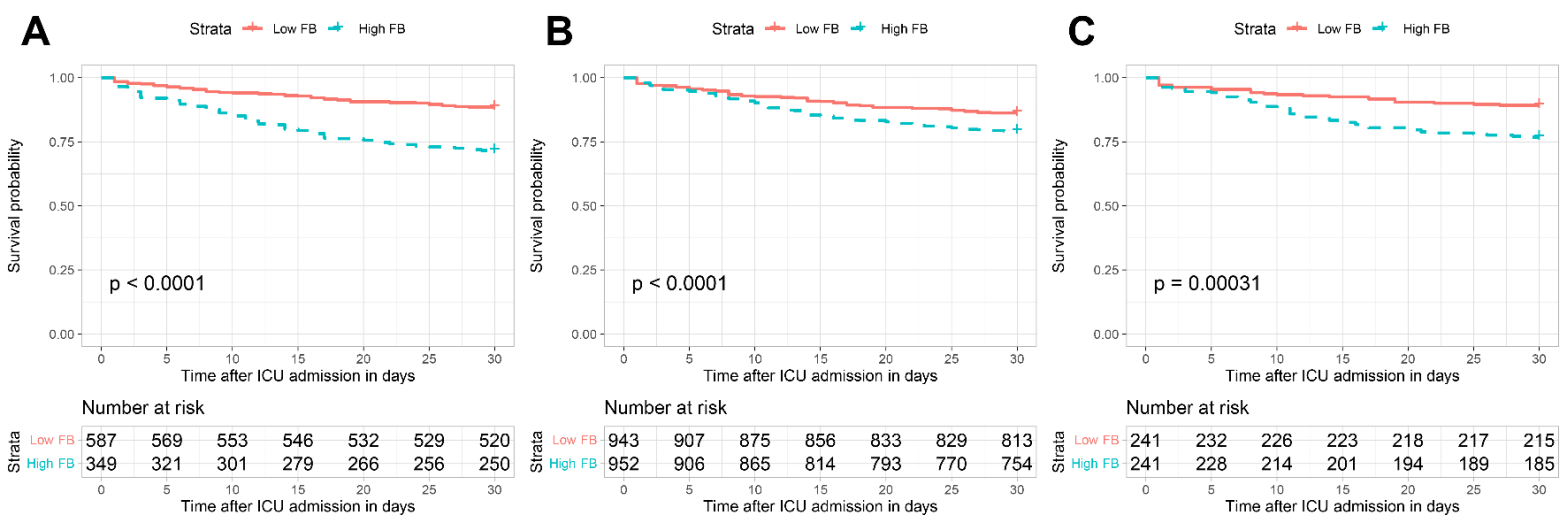
The 30-day cumulative survival probability for each group. (A) Original cohort, (B) After IPTW, (C) After PSM. Abbreviations: FB, fluid balance; IPTW, inverse probability of treatment weighting; PSM, propensity score-matching

In the IPTW model, baseline characteristics between groups were well balanced. After PSM, 241 patients were well matched by a 1:1 ratio for each group. No significant differences were found in the baseline characteristics between the matched groups (Supplement Table S2). Regression analyses verified stronger monotonic associations of FB with in-hospital mortality (IPTW model: OR 1.66, 95% CI 1.39–1.99 per SD increase, p < 0.001; PSM model: OR 1.45, 95% CI 1.13–1.88 per SD increase, p = 0.004) and 30-day mortality (IPTW model: OR 1.67, 95% CI 1.40–1.99 per SD increase, p < 0.001; PSM model: OR 1.35, 95% CI 1.04–1.74 per SD increase, p = 0.022) (Table 2). In IPTW and PSM models, high FB consistently showed higher in-hospital mortality (IPTW model: OR 1.94, 95% CI 1.52–2.49, p < 0.001; PSM model: OR 2.93, 95% CI 1.75–4.90, p < 0.001) and 30-day mortality (IPTW model: OR 1.65, 95% CI 1.29–2.10, p < 0.001; PSM model: OR 2.50, 95% CI 1.51–4.15, p < 0.001), compared with the low FB group (Table 2). As depicted in the Kaplan-Meier survival curves, patients with low FB show better 30-day survival than those with high FB in the crude model (Fig. 3A), the IPTW model (Fig. 3B), and the PSM model (Fig. 3C).

## Discussion

Currently, few studies have discussed the relationship between FB and mortality in critically ill patients with heart failure and sepsis. To our knowledge, the cut-off value of FB is the first to be reported in this study for critically ill patients with heart failure and sepsis. In this study, fluid intake, fluid output, and FB have been corrected for each patient’s body weight, which is an improvement over many studies that just calculated absolute volumes of fluid resuscitation. We found that high FB was an independent risk factor for in-hospital mortality and 30-day mortality, as well as associated with longer LOS in ICU and hospital. The conclusions are consistent and robust by subgroup, multivariable logistic regression, IPTW, and PSM analyses, which can provide a reliable assessment for the impact of FB on prognosis in such patients in a real clinical setting.

Our results show the benefits of low FB and verify the detrimental impacts of fluid overload. The potential mechanism may be that high FB accelerates the multiple organ disorder [13]. First, cardiotoxicity and glycocalyx damage induced by fluid overload has been confirmed in animal models [14, 15]. Large volume intake is a contributor to myocardial injury because of myocardial edema and oxidative stress. The FEAST trial showed cardiovascular collapse generated refractory shock, contributing most to the excessive mortality [16]. Second, for patients with high FB, increased atrial and venous pressure can facilitate the fluid transfer to the interstitial space and aggravate tissue edema, which results in tissue distortion and microcirculation disorder, and thus leads to cell metabolism disturbance [13, 17]. Third, increased venous pressure can minimize renal perfusion, which is harmful to kidney function [17]. Fourth, aggressive fluid intake is proved to promote intra-abdominal hypertension, which is closely related to respiratory dysfunction, reduced hepatic perfusion, bowel malabsorption and obstruction, and even death [18, 19]. On the other hand, although no statistical differences were found in the subgroup of patients with COPD or cirrhosis, the small sample size of each stratification limits its results. Besides, FB status also depends on the previous extent of fluid accumulation, although patients who can achieve low or negative FB may indicate their better organ function [20]. Further studies are needed to investigate the specific mechanism.

Early and immediate fluid resuscitation has been recommended in the updated Surviving Sepsis Campaign guidelines because fluid intake can reverse hypotension and correct tachycardia in patients with sepsis [5, 13]. However, this guideline also acknowledged that this recommendation is based on low-quality evidence [21]. Several studies reported the relationship between FB and mortality in sepsis by setting various thresholds of volume and time points. A multicentric Scandinavian study showed no statistical difference between FB and mortality [22]. Moreover, a meta-analysis published by Meyhoff et al. did not find the protective effects of restrictive fluid intake as well [23]. On the contrary, there is still evidence of harmful effects of fluid resuscitation [24, 25]. Fluid intake of more than 5 L on the 1st day of hospital admission increased the risk of excess deaths [26]. Similarly, FB over 3 L between the 24th and the 48th hour after diagnosis increased the risk of mortality in severe sepsis [27]. Consecutive positive FB within 3 days had a higher risk of in-hospital mortality [28]. However, the time point of FB varies among current studies and no unified criteria have been achieved. Conclusions regarding the relationship between early FB status and mortality are conflicting in current studies [24, 29]. It has also been suggested that early fluid accumulation (within 24 h after ICU admission) was not associated with increased mortality in septic patients [24, 29], and even Brotfain et al. highlighted the beneficial effects of early fluid resuscitation [30]. This might indicate that septic patients have a certain tolerance for the adverse effects of fluid overload, and furthermore, early fluid resuscitation is still recommended by the guidelines [31]. Besides, the volume of fluid intake and output may be recorded inaccurately during clinical practice due that partial fluid losses are difficult to measure. Many studies only calculated the absolute volumes of fluid intake, instead of the relative volumes adjusted by individuals’ weight. Additionally, fluid responsiveness and levels of haemodynamic disturbance are hard to be defined and stratified in various groups [32]. Therefore, with respect to fluid resuscitation for sepsis, the evidence of most trials was not strong enough to make a clear consensus.

As is well-known, limiting fluid intake and increasing fluid output is the principle of heart failure treatment, which is paradoxical with medical treatment for sepsis [7]. Koell et al. noted that fluid overload was associated with adverse prognosis in patients with heart failure and preserved ejection fraction [33]. Fluid accumulation and redistribution result in the expansion of interstitial and intravascular space in organs. Right heart hemodynamic data lack reliability for precise fluid management because of its provided limited information, although commonly utilized for the evaluation of fluid balance status in ICU [6, 34]. Regarding studies on heart failure with sepsis, it has been reported that almost 25% of heart failure patients died of sepsis [35]. The survivors accepted more fluid intake within the first 6 h for patients with heart failure with reduced ejection fraction and septic shock [36].

On the basis of current researches combined with our results, early fluid resuscitation can correct hypotension and increase cardiac output, while late fluid overload by continuous volume intake is harmful. We resume that FB status should be paid attention and the strategy of limited fluid intake should be adopted as long as vital signs have been corrected and reached stability. The cut-off value of FB, instead of positive or negative FB, may provide guidance for the assessment of patients’ states and prognosis, and timely adjust the treatment strategy. The speed and amount of fluid intake should be cautiously treated in patients with heart failure and sepsis.

## Limitations

Several limitations should be considered for our study. First, potential bias may not fully eliminate and undetected confounding factors might exist although subgroup analyses, as well as adjusted, IPTW, and PSM models reached consistent results. Second, fluid overload, as a haemodynamic state, cannot be prospectively randomized so it has to be replaced by FB. FB and haemodynamic status before ICU admission were not available in the database, to a certain extent, which could cause information bias. Third, only the relationship between high FB and in-hospital mortality can be inferred due to the nature of the retrospective study design. Prospective cohort studies are needed to further detect whether a causal association of high FB and mortality exists. In addition, some variables were not included due to excessive missing values. In this study, the relatively small percentage of missing data might also have potential impacts on our results. Multicentric cohort studies are needed to further validate the results due to the single-center study design.

## Conclusion

In conclusion, for critically ill patients with heart failure and sepsis, high FB within the first 24 h after ICU admission could serve as an independent risk factor for in-hospital mortality and 30-day mortality. The avoidance of fluid overload exerts important effects on reducing mortality in such patients. Future studies are needed to further establish the optimal strategy on fluid status management for patients with heart failure and sepsis.

## Electronic supplementary material

Below is the link to the electronic supplementary material.

Supplementary Material 1

Supplementary Material 2

Supplementary Material 3

Supplementary Material 4

Supplementary Material 5

## Data Availability

The datasets generated and/or analyzed during the current study are available in the PhysioBank repository (mimic.physionet.org).

## Abbreviations and acronyms

ACEI: Angiotensin-converting enzyme inhibitor.
ARB: Angiotensin receptor blocker.
CI: Confidence interval.
COPD: Chronic obstructive pulmonary disease.
CKD: Chronic kidney disease.
FB: Fluid balance.
ICU: Intensive care unit.
IPTW: Inverse probability of treatment weighting.
IQR: Interquartile range.
LOS: Length of stay.
LVEF: Left ventricular ejection fraction.
MAP: Mean arterial pressure.
MIMIC: Medical Information Mart for Intensive Care.
NT-proBNP: N-terminal pro-B-type natriuretic peptide.
OR: Odds ratio.
PSM: Propensity score matching.
SD: Standard deviation.
SOFA: Sequential organ failure assessment.
