# Supplementary figures and images for: Association between fluid balance and mortality for heart failure and sepsis: a propensity score-matching analysis
Source: BMC Anesthesiol. 2022 Oct 22;22:324. doi: 10.1186/s12871-022-01865-5 (PMC9587660; doi:10.1186/s12871-022-01865-5)

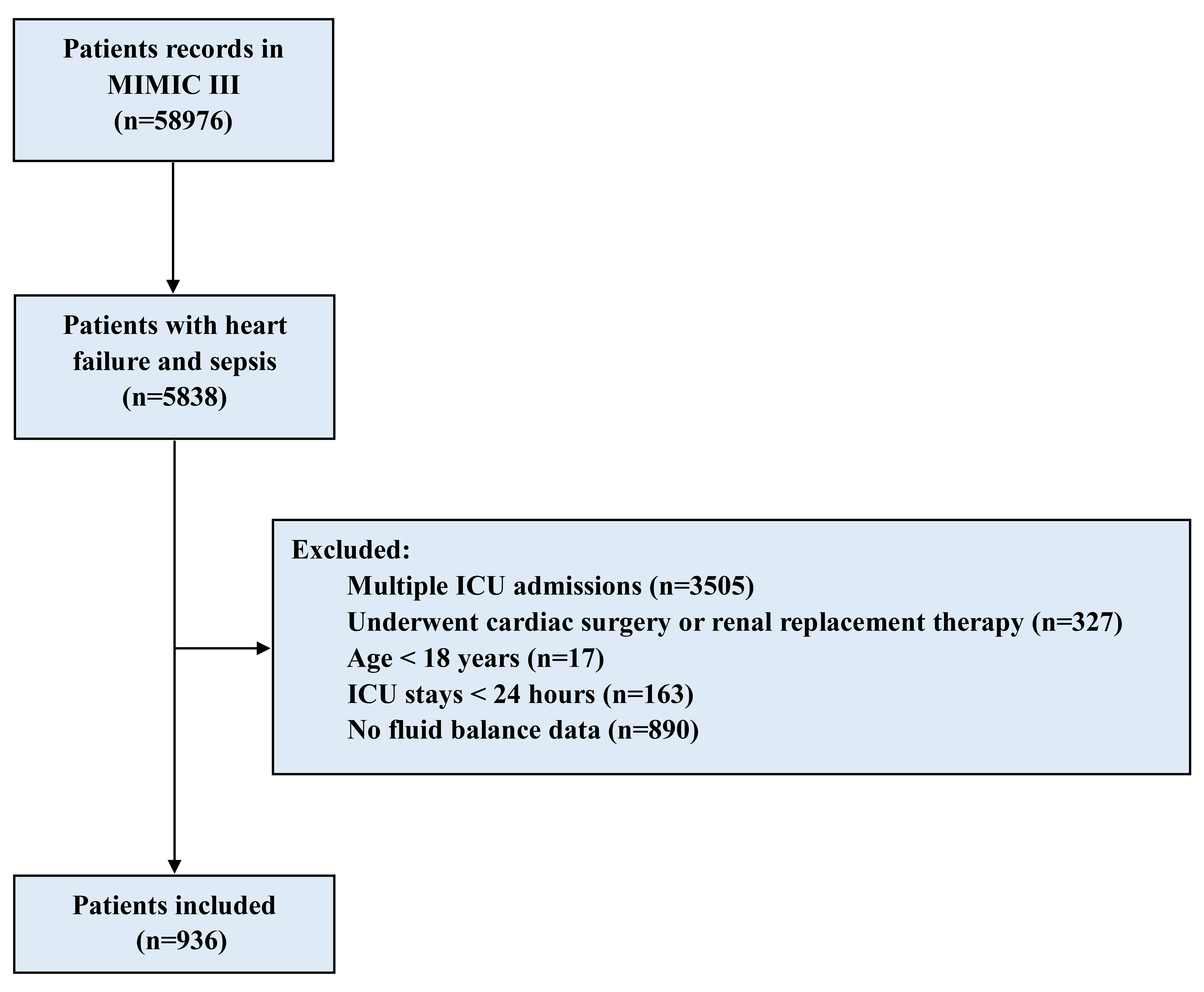

Supplement: Supplementary file 1 — Supplementary Material 1 [file 12871_2022_1865_MOESM1_ESM.tif]
