# Supplementary material for: Association between fluid balance and mortality for heart failure and sepsis: a propensity score-matching analysis
Source: BMC Anesthesiol. 2022 Oct 22;22:324. doi: 10.1186/s12871-022-01865-5 (PMC9587660; doi:10.1186/s12871-022-01865-5)
Supplement: Supplementary file 2 — Supplementary Material 2 [file 12871_2022_1865_MOESM2_ESM.docx]

Supplementary material:

Figure S1. Study flow chart.

Table S1. Missing number and percentage for variables.

Table S2. Comparisons of the covariates between high and low FB groups in IPTW and PSM models.
