# Supplementary material for: Association between fluid balance and mortality for heart failure and sepsis: a propensity score-matching analysis
Source: BMC Anesthesiol. 2022 Oct 22;22:324. doi: 10.1186/s12871-022-01865-5 (PMC9587660; doi:10.1186/s12871-022-01865-5)
Supplement: Supplementary file 3 — Supplementary Material 3 [file 12871_2022_1865_MOESM3_ESM.docx]

Table S1. Missing number and percentage for variables.

| Variables | Missing number (%) |
| --- | --- |
| Age (years) | 0 (0) |
| Male, n (%) | 0 (0) |
| Weight (kg) | 0 (0) |
| Ethnicity, n (%) | 0 (0) |
| Infection site, n (%) | 0 (0) |
| LVEF, n (%) | 145 (15.49) |
| MAP (mm Hg) | 45 (4.81) |
| Laboratory |  |
| White blood cell (10^9^/L) | 0 (0) |
| Hemoglobin (g/dL) | 0 (0) |
| pH | 62 (6.62) |
| Serum potassium (mmol/L) | 0 (0) |
| Serum sodium (mmol/L) | 0 (0) |
| Serum bicarbonate (mmol/L) | 0 (0) |
| Serum chloride (mmol/L) | 0 (0) |
| Serum lactate (mmol/L) | 18 (1.92) |
| Serum creatinine (mg/dL) | 0 (0) |
| Troponin T (ng/mL) | 151 (16.13) |
| NT-proBNP (pg/mL) | 237 (25.32) |
| Drug |  |
| ACEI/ARB, n (%) | 0 (0) |
| Beta-blocker, n (%) | 0 (0) |
| Vasopressor, n (%) | 0 (0) |
| SOFA | 0 (0) |
| Comorbidities, n (%) |  |
| Coronary heart disease | 0 (0) |
| Hypertension | 0 (0) |
| COPD | 0 (0) |
| CKD | 0 (0) |
| Cirrhosis | 0 (0) |
| Diabetes | 0 (0) |
| Fluid balance status |  |
| Fluid intake (ml/kg/24h) | 0 (0) |
| Fluid output (ml/kg/24h) | 0 (0) |
| Fluid balance (ml/kg/24h) | 0 (0) |
| Clinical Outcomes |  |
| In-hospital mortality, n (%) | 0 (0) |
| 30-day mortality, n (%) | 0 (0) |
| ICU LOS (days) | 0 (0) |
| Hospital LOS (days) | 0 (0) |

Abbreviations: ACEI, angiotensin-converting enzyme inhibitor; ARB, angiotensin receptor blocker; CKD, chronic kidney disease; COPD, chronic obstructive pulmonary disease; ICU, intensive care unit; LOS, length of stay; LVEF, left ventricular ejection fraction; MAP, mean arterial pressure; NT-proBNP, N-terminal pro-B-type natriuretic peptide; SOFA, sequential organ failure assessment.
