# Supplementary material for: Association between fluid balance and mortality for heart failure and sepsis: a propensity score-matching analysis
Source: BMC Anesthesiol. 2022 Oct 22;22:324. doi: 10.1186/s12871-022-01865-5 (PMC9587660; doi:10.1186/s12871-022-01865-5)
Supplement: Supplementary file 4 — Supplementary Material 4 [file 12871_2022_1865_MOESM4_ESM.docx]

Table S2. Comparisons of the covariates between high and low FB groups in the IPTW and PSM models.

| Variables | IPTW Model | | p | PSM Model | | p |
| --- | --- | --- | --- | --- | --- | --- |
|  | High FB | Low FB |  | High FB | Low FB |  |
| N | 952 | 942.62 |  | 241 | 241 |  |
| Age (years) | 69.45±16.53 | 71.39±14.11 | 0.350 | 72.29±15.09 | 70.28±14.93 | 0.142 |
| Gender, male, n (%) | 565.2 (59.4) | 503.3 (53.4) | 0.193 | 122 (50.62) | 124 (51.45) | 0.855 |
| Weight (kg) | 82.14±23.06 | 81.15±23.44 | 0.733 | 76.44±21.20 | 76.85±18.00 | 0.817 |
| Ethnicity, n (%) |  |  | 0.364 |  |  | 0.719 |
| White | 699.5 (73.5) | 714.9 (75.8) |  | 185 (76.76) | 183 (75.93) |  |
| Black | 71.2 (7.5) | 93.1 (9.9) |  | 22 (9.13) | 27 (11.2) |  |
| Other | 181.3 (19.0) | 134.6 (14.3) |  | 34 (14.11) | 31 (12.86) |  |
| Infection site, n (%) |  |  | 0.372 |  |  | 0.574 |
| Blood | 704.5 (74.0) | 651.5 (69.1) |  | 168 (69.71) | 158 (65.56) |  |
| Urine | 157.4 (16.5) | 196.8 (20.9) |  | 48 (19.92) | 52 (21.58) |  |
| Other | 90 (9.5) | 94.3 (10.0) |  | 25 (10.37) | 31 (12.86) |  |
| LVEF, n (%) |  |  | 0.861 |  |  | 0.602 |
| ≤ 40 % | 247.7 (26.0) | 249.7 (26.5) |  | 64 (26.56) | 55 (22.82) |  |
| 40 - 50 % | 104.9 (11.0) | 88.4 (9.4) |  | 20 (8.3) | 19 (7.88) |  |
| ≥ 50 % | 599.4 (63.0) | 604.5 (64.1) |  | 157 (65.15) | 167 (69.29) |  |
| MAP (mm Hg) | 77.38±17.82 | 77.33±17.85 | 0.978 | 75.93±17.31 | 76.17±18.53 | 0.885 |
| Laboratory test |  |  |  |  |  |  |
| White blood cell (10^9^/L) | 10.5 (6.8, 16.3) | 10.2 (7.1, 15.96) | 0.926 | 10.3 (6.7, 15.3) | 9.7 (7.1, 13) | 0.477 |
| Hemoglobin (g/dL) | 11.61±1.94 | 11.52±2.00 | 0.603 | 11.51±2.02 | 11.74±2.07 | 0.220 |
| pH | 7.37±0.10 | 7.36±0.09 | 0.299 | 7.36±0.10 | 7.36±0.09 | 0.642 |
| Serum potassium (mmol/L) | 4.34±0.92 | 4.40±0.81 | 0.441 | 4.41±0.96 | 4.38±0.77 | 0.742 |
| Serum sodium (mmol/L) | 137.03±5.25 | 137.53±4.64 | 0.297 | 137.68±5.41 | 137.74±4.68 | 0.907 |
| Serum bicarbonate (mmol/L) | 24.28±4.91 | 24.33±5.10 | 0.909 | 23.90±4.88 | 24.09±4.81 | 0.659 |
| Serum chloride (mmol/L) | 101.41±6.21 | 101.59±5.91 | 0.722 | 102.15±6.49 | 101.58±5.97 | 0.321 |
| Serum lactate (mmol/L) | 1.9 (1.3, 2.9) | 1.9 (1.3, 2.8) | 0.889 | 2 (1.4, 2.7) | 1.9 (1.4, 2.7) | 0.841 |
| Serum creatinine (mg/dL) | 1.18 (0.9, 1.84) | 1.3 (0.9, 2) | 0.901 | 1.2 (0.9, 2.1) | 1.3 (0.9, 2.1) | 0.540 |
| Troponin T (ng/mL) | 0.04 (0.02, 0.11) | 0.04 (0.02, 0.11) | 0.666 | 0.04 (0.02, 0.08) | 0.04 (0.01, 0.1) | 0.257 |
| NT-proBNP (pg/mL) | 5749 (2006, 13255) | 4399 (1869, 12980) | 0.841 | 5932 (2214, 11956) | 5040 (1869, 13621) | 0.539 |
| Drug, n (%) |  |  |  |  |  |  |
| ACEI/ARB | 123.1 (12.9) | 110.6 (11.7) | 0.814 | 13 (5.39) | 15 (6.22) | 0.697 |
| Beta-blocker | 392.3 (41.2) | 400.1 (42.4) | 0.797 | 96 (39.83) | 100 (41.49) | 0.711 |
| Vasopressor | 357.2 (37.5) | 375.5 (39.8) | 0.597 | 116 (48.13) | 107 (44.40) | 0.411 |
| SOFA | 5 (3, 8) | 5 (3, 8) | 0.482 | 6 (4, 9) | 5 (3, 9) | 0.342 |
| Comorbidities, n (%) |  |  |  |  |  |  |
| Coronary heart disease | 434.0 (45.6) | 435.5 (46.2) | 0.899 | 106 (43.98) | 114 (47.3) | 0.464 |
| Hypertension | 460.1 (48.3) | 493.6 (52.4) | 0.410 | 127 (52.7) | 140 (58.09) | 0.234 |
| COPD | 63.8 (6.7) | 72.1 (7.6) | 0.723 | 12 (4.98) | 17 (7.05) | 0.338 |
| CKD | 311.5 (32.7) | 332.0 (35.2) | 0.564 | 80 (33.2) | 79 (32.78) | 0.923 |
| Cirrhosis | 80.1 (8.4) | 82.8 (8.8) | 0.889 | 20 (8.3) | 21 (8.71) | 0.870 |
| Diabetes | 413.0 (43.4) | 439.3 (46.6) | 0.505 | 102 (42.32) | 105 (43.57) | 0.783 |

Abbreviations: ACEI, angiotensin-converting enzyme inhibitor; ARB, angiotensin receptor blocker; CKD, chronic kidney disease; COPD, chronic obstructive pulmonary disease; FB, fluid balance; LVEF, left ventricular ejection fraction; IPTW, inverse probability of treatment weighting; MAP, mean arterial pressure; NT-proBNP, N-terminal pro-B-type natriuretic peptide; PSM, propensity score-matching; SOFA, sequential organ failure assessment.
